# Monitoring the Quality of Medicines: Results from Africa, Asia, and South America

**DOI:** 10.4269/ajtmh.14-0535

**Published:** 2015-06-03

**Authors:** Mustapha Hajjou, Laura Krech, Christi Lane-Barlow, Lukas Roth, Victor S. Pribluda, Souly Phanouvong, Latifa El-Hadri, Lawrence Evans, Christopher Raymond, Elaine Yuan, Lang Siv, Tuan-Anh Vuong, Kwasi Poku Boateng, Regina Okafor, Kennedy M. Chibwe, Patrick H. Lukulay

**Affiliations:** Promoting the Quality of Medicines Program, U.S. Pharmacopeial Convention, Rockville, Maryland

## Abstract

Monitoring the quality of medicines plays a crucial role in an integrated medicines quality assurance system. In a publicly available medicines quality database (MQDB), the U.S. Pharmacopeial Convention (USP) reports results of data collected from medicines quality monitoring (MQM) activities spanning the period of 2003–2013 in 17 countries of Africa, Asia, and South America. The MQDB contains information on 15,063 samples collected and tested using Minilab^®^ screening methods and/or pharmacopeial methods. Approximately 71% of the samples reported came from Asia, 23% from Africa, and 6% from South America. The samples collected and tested include mainly antibiotic, antimalarial, and antituberculosis medicines. A total of 848 samples, representing 5.6% of total samples, failed the quality test. The failure proportion per region was 11.5%, 10.4%, and 2.9% for South America, Africa, and Asia, respectively. Eighty-one counterfeit medicines were reported, 86.4% of which were found in Asia and 13.6% in Africa. Additional analysis of the data shows the distribution of poor-quality medicines per region and by therapeutic indication as well as possible trends of counterfeit medicines.

## Introduction

The spread of substandard, spurious, falsely labeled, falsified, or counterfeit (SSFFC) medicines has been a global concern. Their prevalence increasingly threatens public health by jeopardizing patient safety, leading to treatment failure, contributing to development of drug resistance, and possibly leading to diminished confidence in health systems.^1^,^2^

Protecting the public from exposure to SSFFC medicines (also referred to as poor-quality medicines in this manuscript) requires the presence of a robust medicines quality assurance system. Establishing such a system can be challenging in resource-limited countries, as they are more vulnerable to trafficking of poor-quality medicines, which gives rise to potentially devastating effects.^3^,^4^ In addition to good medicines quality assurance systems, transparent access to reliable data on the quality of medicines is needed from country authorities, and most importantly, these data need to be shared with neighboring countries as well as regional and global initiatives to combat the spread of SSFFC medicines. The availability of such data can help put in place informed and adequate strategies to reduce the prevalence of poor-quality medicines. Although most publicly available reports on poor-quality medicines are from independent studies conducted in some countries,^5^–^9^ there is a paucity of such data, despite the prevalence of SSFFC medicines. To address the deficit of reliable data on medicines quality, in 2010, the Promoting the Quality of Medicines (PQM) program—funded by the United States Agency for International Development (USAID) and implemented by the U.S. Pharmacopeial Convention (USP)—publicly released the Medicines Quality Database (MQDB).^10^

MQDB is a freely accessible, online tool that tracks medicines tested for quality in selected countries in Africa, Asia, and South America. The database is available at http://www.usp.org/worldwide/medQualityDatabase/.^11^

The data available in MQDB were generated during post-marketing surveillance of medicines quality in these countries following similar protocol for sample collection, screening, testing, and reporting. The testing involves field screening tests, verification tests to confirm results obtained in the field by repeating a screening test on failed and doubtful samples, and a pharmacopeial or validated method to confirm the results of the verification test. This methodology is referred to as the three-level approach for monitoring the quality of medicines available in the market.^12^

In this paper, data currently available in MQDB, plus new data not yet publicly available, were analyzed with a focus on global and regional data, and the distribution of therapeutic classes, their failure proportions, the presence of counterfeit medicines, and possible trends were explored. Three out of the 17 total countries examined for this paper do not publicly release their results in the database; however, their data were included in the global and regional analyses.

## Materials and Methods

The routine sampling of a variety of medicines, including antimalarial, antibiotic, antiretroviral, antituberculosis, and other essential medicines from the World Health Organization (WHO) Model List of Essential Medicines^13^ is conducted by government-trained staff from participating countries. It takes a nonrandom, convenience sampling approach with sample types and collection volume subject to available funding and regulatory focus at the time. In some cases, however, a focus on high-risk areas and/or specific types of medicines may be targeted when there is new on-the-ground information indicating the possibility of quality problems.^14^

The medicines were collected from public, private, and informal sector outlets. The public sector is defined as government institutions. The private sector is defined as for-profit, licensed establishments that dispense and/or commercialize medicines. The informal sector is defined as unregulated for-profit establishments and vendors that operate without a license to sell medicines.

These data cannot be considered globally, or regionally representative; however, analyses of the database as a whole, or broken down by country and region, can provide general guidance on trends of poor-quality anti-infective medicines.

In general, a minimum of 50 units and a maximum of 100 units per sample are needed to conduct screening, verification, and confirmatory testing. If the sampling team is unable to collect the minimum number of units, the protocol indicates to collect what is available. Collecting insufficient sample, however, may limit the ability to conduct confirmatory testing in the future.

Standardized guidelines and reporting tools for visual inspection, screening tests, verification testing, compendial testing, and reporting of results are used by all countries reporting to the database. The screening tests involve visual inspection, simple disintegration, and thin layer chromatography (TLC) using Global Pharma Health Foundation (GPHF) Minilab^®^ kits at sentinel sites.^15^ The Minilab is a rugged, portable laboratory, designed for field-use, which contains the means to perform simple tests of rapid medicines quality verification and counterfeit medicines detection for 70 types of medicines. Visual inspection includes close examination of the tablet, capsule or suspension appearance as well as the condition of the primary and secondary packaging, information on the label including in-country registration number (if applicable), and examination of the informational insert for patients.

The results of testing are reported to the national regulatory body, as well as to the MQDB. The relevant authorities of all participating countries approved the public dissemination of information in this database.

A sample reported as failed in MQDB could be substandard, counterfeit, expired, or have failed visual inspection. Samples that fail visual inspection due to expiry or lack of registration are not sent for confirmatory quality testing and are counted as failed without additional review. For a sample to fail, after passing visual inspection, it has 1) failed Minilab testing (simple disintegration + TLC), 2) failed verification of these tests, and/or 3) failed confirmatory testing in a qualified laboratory (usually the country’s national quality control laboratory). Reasons for failure include absence of active pharmaceutical ingredient (API); presence of too much or too little API; presence of the wrong API; failed assay, dissolution, or related compounds/impurity tests. Samples that failed only at the Minilab level, and were not verified and confirmed, were not included in this analysis; however, they are included in the overall sample numbers.

According to the standardized three-level approach, after visual inspection, the sample undergoes simple disintegration and TLC tests using the Minilab. If a sample passes these screening tests, it is normally considered as “passed” unless the protocol requires all samples to go through further testing regardless of Minilab results.

The definitions of substandard and counterfeit used in MQDB are essentially the same as the WHO SSFFC definitions, which are as follows:

Substandard—Noncompliant sample that failed visual inspection, Minilab, verification, or confirmatory testing due to too much/too little API, or failed dissolution and/or impurity testing; expired products and non-registered products are also considered substandard.

Counterfeit—A product with either the wrong active ingredient or no active ingredient.† †Not all countries adhere to the WHO SSFFC definition of “counterfeit,” however. For example, in some countries, a medicine containing less than 80% or more than 120% of the API claimed on the label is considered counterfeit.

There were also unexpired samples that failed Minilab testing, but were not sent for confirmatory testing for the following reasons:

1.  Verification or confirmatory testing was delayed until after sample expiration due to funding or other management issues;
2.  Insufficient units were collected due to limited available stock, and without sufficient volume of sample, confirmatory testing cannot be performed;
3.  No monograph or in-house validation method was available to test the medicine collected; or
4.  Various others, such as a lack of reagents, reference standards, and/or necessary testing equipment.

These samples were not classified as failed, because they did not receive confirmatory testing as required.

PQM supports the medicine regulatory authority (MRA) of each country to efficiently register all new medicines.^16^ PQM also provides the initial and follow-up trainings to local country staff on the proper use of the Minilab for performing screening tests and developing sampling strategies and plans, including the selection of medicines and areas of sampling. The sampling of medicines takes place at the central as well as peripheral level, including remote areas, called sentinel sites. A sentinel site may refer to a location where the Minilab is based, which could be a city or department/region.

A global and regional analysis was performed on all medicines collected and tested from 2003 to 2012, using the data that are publicly available in MQDB with the addition of unpublished information detailing reasons for failure, and additional data from 2013, which has not yet been publically released. The study team collated all results using Microsoft Excel 2010 to produce descriptive statistics.

## Results

### Distribution of samples.

For the period of 2003–2013, data were compiled on 15,063 medicine samples collected and tested in 17 countries throughout Africa,‡ Asia,¶ and South America.§ As shown in Table 1, data collected during the period 2003–2007 were almost exclusively from Asia. Submission of data from medicines quality monitoring (MQM) activities in South America and Africa began in 2008 and 2009, respectively. None of the 17 countries, with the exception of Cambodia, have consistently collected and tested medicines between 2003 and 2013. Thailand and Peru were the least consistent in reporting data to MQDB. ‡Ghana, Ethiopia, Liberia, Kenya, and Mozambique.
¶Cambodia, Indonesia, Laos, Myanmar, Philippines, Thailand, Vietnam, China (Yunnan Province).
§Colombia, Ecuador, Guyana, Peru.

Figure 1

**Figure 1.**
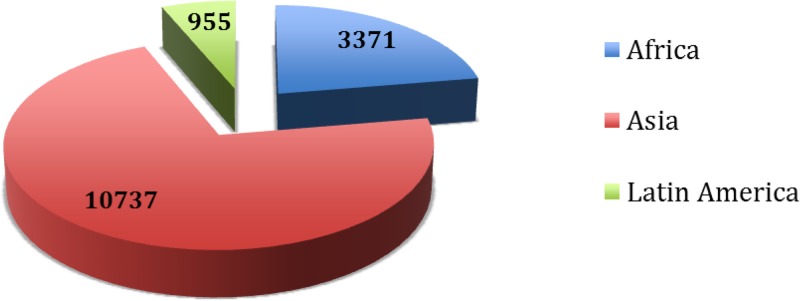
Distribution of samples by region.

and Table 2 show the distribution of data spanning the period of 2003–2013 by region. Of the total 15,063 samples, 71.3% were from Asia, 22.4% from Africa, and 6.3% from South America. As shown in Figure 2

**Figure 2.**
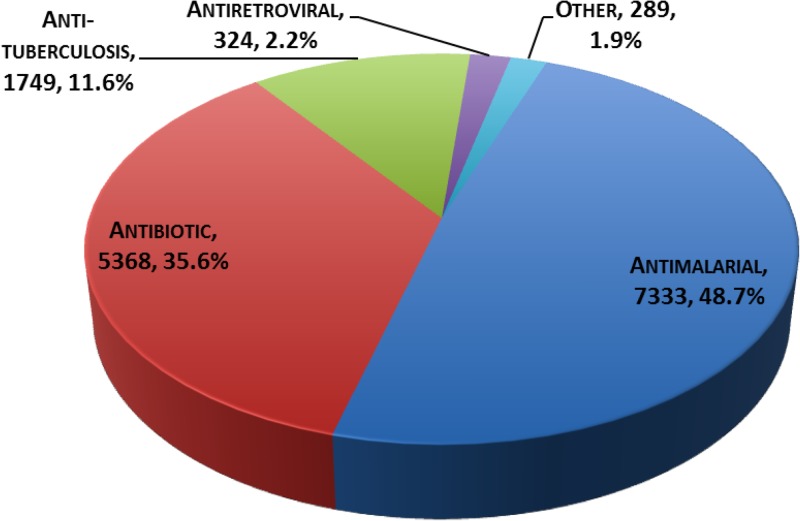
Distribution of samples by therapeutic indication.

, antimalarial medicines represent about half of the entries in the database.

### Failure proportions and distribution.

A total of 848 samples failed quality testing at the screening level, or during verification and/or confirmatory testing. The samples represent a 5.6% failure rate for all 15,063 samples reported in the database. As shown in Table 3, a total of 81 samples were identified as counterfeit, representing 9.6% of total failed samples, and the remaining 767 failed samples were classified as substandard (90.4%). This set of substandard samples includes 231 samples that failed screening tests using the Minilab without undergoing confirmatory testing (failed visual inspection or expired when collected or not registered in country). By region, overall, South America reported an 11.5% failure rate, followed by Africa at 10.7%, and Asia at 3.5%.

A closer look at the distribution of failed samples by therapeutic indication, in Table 4, revealed that the number of failed antimalarial medicines was the highest at 56.4% of the failed samples; however, proportionate to the samples of antimalarials collected, they represent a failure rate of 6.5%. The category “Other” includes anti-inflammatory, analgesic, antihistaminic, antihypertensive, and antifungal medicines as well as medicines for maternal and child health, and vitamins. The Other category represented 10.6% of the failed samples, and the failure rate for this combined group was 18.6%. For approximately 1.5% of failed samples, complete information about the API was not reported to the database.

Table 5 shows the regional distribution of failed samples by therapeutic indication. In Africa, the highest failure rate was reported for antituberculosis medicines (66.2%) and in Asia, antimalarial medicines had the highest rate of failed samples (5.8%) (combined category of “other” excluded). In South America, antibiotics had the highest rate of failure, with 20.9% of samples failing, with antituberculosis medicines also reporting a high rate of failure at 15.3% of samples tested. Although the number of antiretroviral samples was relatively low, the African region has the highest number of failures for this therapeutic category. The total number of antiretroviral medicine failures from Africa is 33, but the total number sampled (the denominator) cannot be released in this article, as some African countries declined to make their data publicly available. This is also the case for the failure rates of antituberculosis medicines in Africa: the total number of antituberculosis medicines that failed quality testing is shown, but the denominator is understated.

Globally, the therapeutic indication listed in this paper as “Other” has the highest rate of failure, averaging 18.6% (Table 4). The rate of failure for this category of medicines ranged from 15.3% in Africa to 31% in South America (Table 5). It is of note that in the latter, with the exception of antimalarials, only 19 of 460 samples (4.1%) failed analytical testing, whereas the rest of the failures were due to incomplete or erroneous information provided on the package or insert.

In examining the data by sector, Table 6 shows that the difference between medicine quality failures in the public sector (36.2%) and the private sector (39.3%) is not large. The informal sector accounted for only 1.7% of the failures.

The authors searched MQDB for the names of purported manufacturers of poor-quality medicines. The most frequently encountered manufacturers were Brainy Pharmaceuticals, which does not exist as an actual company; Mekophar Chemical Pharmaceutical Joint-Stock Co., Macleods Pharmaceuticals Ltd., Bliss GVS Pharmaceuticals Ltd., Matrix Laboratories Limited, and Guilin Pharmaceutical Company Ltd. Numerous poor-quality medicines were found in Asia from Brainy Pharmaceuticals. The name of the manufacturer or the brand or commercial name of a medicine, as it appears in the MQDB, is the name claimed on the label; however, the manufacturer’s name on the label may not be the true source of the tested sample. For example, fake artesunate found in Asia was falsely labeled with Guilin Pharmaceuticals Ltd. as the manufacturer, yet further investigation showed that this company was not the true source of the product. Several failed samples did not have any information on the manufacturer or the country of origin.

### Substandard products.

As indicated above, a total of 767 samples were substandard. Table 3 indicates geographical distribution of substandard and counterfeit products, which suggests higher numbers in Africa and Asia. As shown in Table 2, however, the proportion of substandard medicines was highest in South America with 11.5%, followed by Africa (10.4%) and Asia (2.9%). The distribution of substandard medicines by therapeutic indication, as shown in Table 7, indicates that antimalarial (52.5%), antibiotic (18.9%), and antituberculosis (12.4%) medicines had the highest occurrence of all substandard medicines found in the database.

### Counterfeit products.

In the database, a total of 81 products were classified as counterfeit. The highest number of counterfeit medicines was found in Asia with a total number of 70 samples, representing 86% of counterfeit products (Table 3). All remaining counterfeit medicines, 11 in total, were found in Africa. No counterfeit medicines were found from participating countries in South America.

The most frequently counterfeited products identified in the database were antimalarial medicines with 75 samples, representing 92.6% of all counterfeit medicines. Table 8 shows the distribution of all counterfeit medicines by API. Artesunate has the highest frequency of counterfeits with 35 incidences (43.2%), followed by tetracycline (listed as antimalarial in the database), quinine sulfate, and chloroquine (12.3% each).

**Figure 3.**
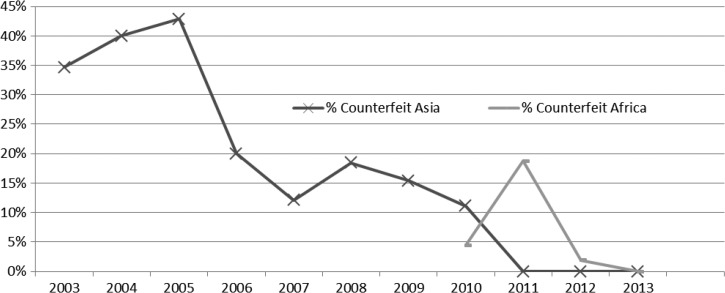
Trends in the proportion of counterfeit medicines during the years 2003–2013. Data from 2014 have not been received from the majority of countries; as of July 2014, no counterfeits had been reported.

The trend of counterfeit medicines reported in the database was analyzed. As depicted in Figure 3

, the percent of failed samples that were counterfeit decreased during the period from 2005 to 2011 in Asia, and no counterfeit medicines from the region have been reported since that time. The occurrence of counterfeit medicines in Africa was reported in the years 2010–2012, but no clear trend is yet evident. Most of the medicines from South America included in MQDB were sampled in the public sector. In general, the public sector is more strongly regulated than the private sector, and this may be one of the reasons why the public sector had fewer counterfeits found.

## Discussion

Analysis of data obtained from MQM activities and listed in MQDB showed a continuous flow of results from participating countries in Asia and Africa. The number of participating countries increased starting in 2008, likely reflecting greater support of MQM activities in these regions. Antimalarials make up the bulk of samples in the database due to the funding sources supporting sample collection. The number of failed samples was highest for antimalarial medicines as a result of their prevalence in the database. MQM activities that generated data reported during the period 2003–2007 were almost exclusively conducted in Asia. This factor is reflected by the high number of samples from Asia reported through 2013, representing more than 70% of the total data and with a combined failure rate of 3.5%. The number of samples reported from participating countries in South America was the lowest, representing only 6.3%; however, the proportion of poor-quality medicines was highest in this region at 11.5% of samples tested.

The samples listed in this paper under the therapeutic indication “other” had the highest proportion of failure. Although this group of therapeutic indications represented only 3.2% of samples reported, the failure proportion was 18.6%, representing 10.6% of total failed samples. Sentinel site teams sample medicines outside the core therapeutic areas when they are identified as suspicious, leading to an increased rate of failure in this category.

The data available in the MQDB give important insight into the quality of medicines in the regions reporting to the database and provides a valuable source for regional and temporal comparisons. Support is needed to implement robust post-marketing surveillance systems covering a wide range of medicines in addition to antimalarial, antituberculosis, and antibiotic medicines since substandard quality is not limited to those categories. Only through continued investment in monitoring, awareness, and enforcement, it will be possible to reduce exposure of the public to poor-quality medicines.

USAID, the President’s Malaria Initiative (PMI), and the President’s Emergency Plan for AIDS Relief (PEPFAR) have supported the strengthening of health systems in many countries around the globe. Their support has helped build medicines quality assurance systems by strengthening the capacity of medicines regulatory authorities and national quality control laboratories as well as improving the management of the supply chains in those countries. Programs for monitoring the quality of medicines have been established through USAID, PMI, and/or PEPFAR funding to periodically control the quality of medicines available to patients. These programs rely on the use of tools that allow screening of a large number of samples at a reduced cost. Such programs have assisted countries in generating evidence-based data on the quality of medicines and have helped develop strategies to counter the spread of poor-quality medicines. MRAs were encouraged to take actions whenever poor-quality medicines were found.

The data analyzed in this article suggest that the proportion of poor-quality medicines that were found in Asia to be counterfeit has decreased from a peak of over 40% of failed samples in 2005 to current low levels. To clarify, this statement does not include all medicines throughout Asia; rather, it refers to the anti-infective medicines collected in the participating countries, particularly antibiotics and antimalarials. The decrease in counterfeit medicines may be attributed in part to corrective actions taken by regulatory authorities based on MQM findings and to harsher punishments for counterfeiters and local companies that import or distribute counterfeit medicines.^17^,^18^ There have been numerous successful campaigns to raise awareness about this issue and encourage patients to obtain their medicines from the public sector or a regulated private outlet.^19^,^20^

The present global analysis of data from MQDB provides a snapshot of the quality of antimalarial, antituberculosis, and antibiotic medicines as represented in the database. Several sources of bias exist in the data. First, the sample size required for verification and confirmatory testing is often too large for smaller private outlets, leading failures from the private sector to be dropped from the analysis. Second, delays in confirmatory testing due to central laboratory capacity reduce the number of confirmed failures because samples expire before testing. Third, the focus of most sample collection is established outlets, whether public or private, with less regulatory action targeting illegal outlets, where the quality of medicines may be poorer. Outlets selling counterfeit and deliberately substandard medicines are, by nature, clandestine and impermanent, making identification via routine investigations difficult. Finally, donor funding, rather than standard government activities, support sample collection only in therapeutic areas of interest to the donor; as a result, there is no consistent annual cross-sectional collection of drug quality data that could be used to accurately project the prevalence of poor-quality medicines. All these sources of bias serve to reduce the number of confirmed failures which, given greater testing efficiency, expediency, and broader inspection mandates, would be expected. We are unable to provide a precise estimate of the true prevalence of poor-quality and counterfeit medicines circulating in these countries but advise that the relatively high prevalence of poor-quality medicines is understated.

The analysis of MQDB data indicates that poor-quality medicines continue to be identified by regulatory agencies globally, in both the public and private sectors. Government officials and donors should consider support to expand medicines quality initiatives beyond infectious disease treatments to test medicines for chronic diseases, since the epidemiologic transition continues in African and Asian countries and chronic disease increases in prevalence. Technical support for governments and private manufacturers is needed to improve manufacturing quality, registration procedures, and post-marketing safety monitoring to identify manufacturing, supply chain management, and storage conditions, which expose the public to potential harm. On a final note, although it is very important to continue collecting and analyzing MQDB data, it is critical to efficiently communicate the findings to a broad audience. PQM is examining ways to collaborate with WHO’s Rapid Alert System to ensure that verified reports of poor-quality medicines found through country MQM programs are disseminated via the Rapid Alert System and also through regional initiatives (e.g., Association of Southeast Asian Nations) to warn neighboring countries.

## Figures and Tables

**Table 1:** Data submission per country and year

Country	2003	2004	2005	2006	2007	2008	2009	2010	2011	2012	2013
Asia											
Cambodia	X	–	X	X	X	X	B	X	X	X	–
Vietnam	X	X	X	X	X	X	X	X	X	B	B
Laos	X	X	X	X	X	X	X	B	B		
Thailand	–	X	X	–	–	X	X	–	–	B	–
Philippines	–	–	–	–	–	–	X	X	B	B	–
Indonesia	–	–	–	–	–	–	–	–	–	–	B
Myanmar	–	–	–	–	–	–	–	–	–	–	B
China (Yunnan Province)	–	–	X	–	–	–	–	–	–	–	–
Africa											
Ghana	–	–	–	–	–	–	X	X	X	B	B
Kenya	–	–	–	–	–	–	–	X	B	B	–
Mozambique	–	–	–	–	–	–	–	–	–	X	B
Liberia	–	–	–	–	–	–	–	–	–	–	B
Ethiopia	–	–	–	–	–	–	–	–	–	X	B
South America											
Guyana	–	–	–	–	–	X	X	X	X	–	–
Peru	–	–	–	–	–	X	–	X	–	–	–
Colombia	–	–	–	–	–	X	X	X	–	–	–
Ecuador	–	–	–	–	–	–	X	–	–	–	–

B = data submitted and analyzed for this paper; however, as of July 2014, the data were not yet entered into medicines quality database (MQDB), and therefore were not publicly available.

**Table 2:** Prevalence of substandard medicines by region

Region	Total samples	Number of substandard samples	Substandard rate (%)
Africa	3,371	350	10.4
Asia	10,737	307	2.9
South America	955	110	11.5

Number of substandard samples and rate are indicated.

**Table 3:** Regional distribution of failed samples expressed in percent by substandard and counterfeit*

Failure type by region	Number of failed samples	Regional proportion of failure (%)
Substandard (89.6% of failed samples)		
Africa	350	45.6
Asia	307	40.0
South America	110	14.4
Counterfeit (10.4% of failed samples)		
Asia	70	86.4
Africa	11	13.6
Total	848	100

* Substandard—Non-compliant sample that failed visual inspection, Minilab^®^ testing, verification, or confirmatory testing due to too much or too little active pharmaceutical ingredient (API), failed dissolution, and/or failed impurity testing. Expired products and non-registered products are also considered substandard.

Counterfeit—A product with either the wrong active ingredient, or no active ingredient. In some countries, a medicine containing less than 80% or more than 120% of the API claimed on the label is considered counterfeit.

**Table 4:** Distribution of samples and proportion of failure* by therapeutic indications, 2003–2013

Therapeutic indication	Total samples	Number of failed samples (%)
Antimalarial	7,333	478 (6.5)
Antibiotic	5,174	150 (2.9)
Antituberculosis	1,749	95 (5.4)
Antiretroviral	324	35 (10.8)
Others	483	90 (18.6)
Total	15,063	848 (5.6)

* Failed medicines are classified as those that failed visual inspection, were expired at the point of collection, failed verification and/or confirmatory testing. Confirmatory testing failures include failed assay (identity), dissolution, or impurity testing.

**Table 5:** Distribution of failed samples by region and therapeutic indication

Region/therapeutic indication	Number of samples	Number of failed samples (%)	Proportion of failed samples within the region (%)
Antimalarial	2,517	222 (8.8)	61.5
Antibiotic	400	10 (2.5)	2.8
Antituberculosis	74	49 (66.2)	13.6
Antiretroviral	NA	33	NA
Others	307	47 (15.3)	13.0
Total Africa	3,371	361 (10.7)	100
Antimalarial	4,321	249 (5.76)	66.1
Antibiotic	4,473	77 (1.7)	20.4
Antituberculosis	1,616	37 (2.3)	9.8
Antiretroviral	251	2 (0.8)	0.5
Other	76	12 (15.8)	3.2
Total Asia	10,737	377 (3.5)	100
Antimalarial	495	7 (1.4)	6.3
Antibiotic	301	63 (20.9)	57.3
Antituberculosis	59	9 (15.3)	8.2
Other	100	31 (31.0)	28.2
Total South America	955	110 (11.5)	100
Total	15,063	848	5.6

Regional proportion of failure is calculated using the number of failed samples for a therapeutic indication in a region as a percent of the total number of failed samples in the therapeutic indication.

**Table 6:** Distribution of failed samples by sector

Sector	Number of failed samples	Percentage of failed samples
Public	307	36.2
Private	333	39.3
Informal	14	1.7
Unknown	184	21.7
Missing	9	1.1
Quasi-government	1	0.1
Total failed samples	848	100.0

**Table 7:** Substandard medicines distribution by therapeutic indication

Therapeutic indication	Number of failed samples	Total failed samples (%)
Antimalarial	403	52.5
Antibiotic	145	18.9
Antituberculosis	95	12.4
Antiretroviral	35	4.6
Anti-inflammatory	34	4.4
Analgesic	18	2.3
Maternal and child health*	10	1.3
Other	27	2.6
Total	767	100

* Oxytocin and ergometrine.

**Table 8:** Distribution of counterfeit medicines

Medicine	Number of samples	Percentage of total counterfeit
Artesunate	35	43.2
Tetracycline	10	12.3
Quinine Sulfate	10	12.3
Chloroquine	10	12.3
SP*	5	6.2
Mefloquine	4	4.9
Ampicillin	2	2.5
Unknown	1	1.2
Erythromycin	1	1.2
Primaquine	1	1.2
Penicillin	1	1.2
Amoxicillin	1	1.2
Total	81	100

SSFFC = substandard, spurious, falsely labeled, falsified, or counterfeit; WHO = World Health Organization.

Number of counterfeit medicines and percentage of total counterfeit products are provided.

* The combination of both sulfadoxine–pyrimethamine and sulfamethoxypyrazine–pyrimethamine medicines are abbreviated as SP in the table.
